# Statin adherence in patients enrolled in the disease management program for coronary artery disease – comparison between patients’ and general practitioners’ self-reports and patient records

**DOI:** 10.1007/s43999-023-00029-3

**Published:** 2023-09-06

**Authors:** Babak Salam, Anne Schrimpf, Sebastian Münster, Markus Bleckwenn

**Affiliations:** 1 Department of Diagnostic and Interventional Radiology, University Hospital Bonn, Venusberg-Campus 1, 53127 Bonn, Germany; 2 Institute of General Practice, Faculty of Medicine, University of Leipzig, Philipp-Rosenthal-Str. 55, 04103 Leipzig, Germany; 3 Family Practice Dr. Med. Münster, Hermann-Löns-Str. 5, 53840 Troisdorf, Germany

**Keywords:** Disease management program, Adherence, General practitioner, Coronary heart disease

## Abstract

**Introduction:**

Patients with coronary artery disease (CAD) should take a statin daily for secondary prevention. However, statin adherence in patients with CAD is low. This study investigated the proportion of adherent patients enrolled in the disease management program for CAD (DMP-CAD). Adherence was examined by comparing patients’ self-reports, general practitioners’ (GPs) self-reports, and prescription data.

**Methods:**

Between October 2019 and March 2020, all patients enrolled in the DMP-CAD in three GP practices in Germany were invited to participate in the study. Participants completed a questionnaire on the tolerability of statins. Further, prescription data from patient records, low-density lipoprotein (LDL) levels, and GPs’ assessment of statin adherence were examined. The Medication Possession Ratio (MPR) served as a measurement tool for adherence.

**Results:**

Seventy-four patients were included. MPR showed high statin adherence for most patients (83.8%). However, GPs did not reliably identify non-adherence in their patients. Generally, the mean LDL values were above the guideline recommendations (97.7 ± 27.9 mg/dl), with higher values in the non-adherent (123.6 ± 42 mg/dl) than in the adherent group (93.1 ± 22 mg/dl). Non-adherent patients were more likely to be employed (41.7% vs. 11.3%).

**Discussion:**

Patients in this study showed high statin adherence. However, the LDL target value was often not reached. Therefore, GPs should take advantage of the good adherence of their patients and try to lower LDL levels by adjusting the dosage and/or changing the statin prescribed. Future studies should investigate typical characteristics of non-adherent patients in DMP-CAD so that GPs can target these patient groups and improve their adherence.

**Supplementary Information:**

The online version contains supplementary material available at 10.1007/s43999-023-00029-3.

## Introduction

The national health care guideline recommends a continuous statin therapy with a target low-density lipoprotein cholesterol (LDL-C) level of ≤ 70 mg/dl for each patient with coronary artery disease (CAD), regardless of baseline LDL level [1]. For patients with high baseline cardiovascular risk, the new guidelines of the European Societies of Atherosclerosis (EAS) and Cardiology (ESC) recommend an LDL-C value of < 55 mg/dl [2].

The LDL-C level can be controlled by statin prescription. However, studies have shown obstacles to therapy adherence. Approximately only half of all patients with CAD with a regular statin prescription are taking it properly after five years due to common side effects of the medication [3]. Consequently, many patients do not reach the target LDL value [4, 5]. This is attributed to a lack of adherence [6]. Therefore, the national guideline called "Chronic CAD" recommends a review of medication adherence if LDL lowering is not sufficient [1, 5].

Non-adherence can result in health risks for patients with CAD. A study of 59,094 patients new to statins showed that high adherence was associated with a 30% reduced likelihood of acute myocardial infarction (adjusted relative risk 0.63; 95% CI 0.46–0.85) [7]. Other studies showed that good adherence and low LDL were associated with reduced mortality (relative risk reduction of 10% per 1 mmol/l (38.46 mg/dl) reduction in LDL) [8, 9].

In Germany, many patients with CAD are treated by general practitioners (GPs) in a disease management program (DMP). Currently, two million patients with statutory health insurance are enrolled in the DMP-CAD (data from the Federal Joint Committee). However, data on medication adherence of patients in the DMP-CAD are sparse. Recent studies indicated that patients with CAD enrolled in a DMP showed a better general medication adherence compared to non-enrolled patients [10, 11], however, with non-significant small differences for statin adherence [12]. Further, to our knowledge, no study is available to date on GPs’ ability to predict the patients’ statin adherence within the DMP-CAD.

The present study investigated the proportion of statin adherent patients in the DMP-CAD. For this purpose, adherence was examined by comparing patients’ self-reports, GPs’ self-reports, and patient records. Further, patient characteristics that might be associated with limited adherence were investigated.

## Materials and methods

### Recruitment

GP teaching practices at the University of Bonn were contacted via e-mail and telephone (convenience sample). Three out of the 13 contacted practices agreed to participate in the study and were subsequently visited for further information. GP practices were asked to include all DMP-CAD patients in the study who had been taking a statin for at least one year. The only exclusion criterion was the refusal of participation.

### Procedure

The statin adherence of participants was investigated using a self-developed questionnaire in a cross-sectional study. The patients received the questionnaire during their regular DMP consultation. They could answer it in approximately five minutes in the waiting room before the doctor's consultation. Following the consultation, the GP filled out the doctor's part of the questionnaire.

### Structure of the questionnaire

The questionnaire was based on validated questions and parameters of comparable studies on demographic data [13, 14], consumer behavior [15, 16], and medication adherence [17]. In addition, some questions were self-developed. A pre-test of the questionnaire using the think-aloud method was conducted with 16 patients.

The questionnaire is divided into a patient section and a GP section. In addition, data from the patients’ medical records were collected by the first author. The patient part consisted of 18 questions and assessed a) socio-demographic information, b) information on consumer behavior, c) information on medication intake, and d) information on statin tolerance. Education was assessed by using the CASMIN educational classification [14].

In the doctors' part of the questionnaire, the GP used a visual analogue scale (VAS) to indicate the estimated medication adherence of the last 12 months. In addition, the GP was asked whether the patient had already contacted him/her with questions about statin use and whether the patient had already reached the planned LDL target value. If this was not the case, the doctor was asked to indicate the presumed reason for not reaching the LDL target value. The final questionnaire in German and translated into English can be found in Supplementary material S1.

From the patient's record, a) the course of the CAD, b) the LDL serum course, and c) medication adherence to statin use were assessed. To determine adherence, the Medication Possession Ratio (MPR) was used, representing the percentage of days the patient had access to the medication according to prescription data. Following the example of numerous studies on adherence measurement, a cut-off value of < 0.8 was set for non-adherence [17].

### Analysis

All data were analyzed descriptively with SPSS 27 (Armonk, NY, USA) and presented as frequencies. Group differences in categorical variables were analyzed using chi-square or Fisher's exact tests. Estimated effect sizes were reported using Phi (φ) or Cramer's V, depending on the number of categories. Continuous variables were presented as means (M) and standard deviations (SD). Group differences in continuous variables were evaluated with analyses of variance (ANOVAs). The influence of the independent variable "adherence" on dependent variables (age, BMI, number of children, frequency of side effects, pain intensity, last LDL value, target LDL, number of clinical events, GP’s estimation) were investigated. Estimated effect sizes were reported using partial eta squared (η_p_^2^). Only alpha values < 0.05 were considered significant.

### Ethics

The study was approved by the Ethics Committee of the University of Bonn (reference number 276/19). All participating GPs and patients voluntarily participated in the study and gave written informed consent.

## Results

### Sample characteristics

In the participating GP practices, a total of 121 patients enrolled in the DMP-CAD were asked to participate in the study. Forty-seven patients were not included in the survey because they either did not take statins for more than one year (*n* = 16) or refused to participate in the study (*n* = 31). A total of 74 patients were included in the study.

The mean age of the patient participants was 71.1 ± 10.8 years and 67.6% were male. Further, 9.5% of the patients had a nursing degree. Most participants were married (64.9%), had at least one child (86.5%), and were retired (83.8%).

### Health behavior

Most patients (75.7%) were non-smokers. More than half of the patients drank alcohol regularly according to their own statements (60.8%). Most of the alcohol-consuming patients stated that they drank 1–2 glasses/day on average.

### Type of CAD

For 10 participants, no clear information on the type of CAD could be found. We differentiated between interventional (percutaneous coronary intervention (PCI) + coronary artery bypass graft (CABG)) and non-interventional (1-/2-/3-vessel) CAD and found that 66.2% of the participants had interventional CAD. In half of all patients (50%), at least one diagnostic intervention relevant to the coronary artery was documented in the last 12 months.

### Statin use and LDL levels

Most patients (87%) answered the question "Do you take a statin?" with "Yes". The remaining seven patients answered with "I don't know" or "No". Twelve patients suffered from side effects of statin therapy at least once (16.2%). In 75% of the cases, these were reported as muscle complaints. The average severity of side effects was 4.3 ± 1.4 on the numerical rating scale (0 = none to 10 = very strong side effects).

Information on the LDL serum course was found for 72 of 74 participants. The mean LDL value was 97.7 ± 27.9 mg/dl (47—226 mg/dl). In the last 12 months, 16.2% of these patients reached the target LDL value (≤ 70 mg/dl). An additional 17 patients (23%) reached the target LDL at least once during the overall course.

### Statin adherence and GPs’ assessments

MPR was assessed in all 74 participants. The mean value was close to 1 (0.996 ± 0.289). Twelve participants were below the cut-off value of 0.8 and were classified as non-adherent. Thus, the proportion of adherent patients was 83.8%.

The evaluation of the GP's assessment of patient adherence resulted in a mean value of 8.3 ± 1.6 on a Likert scale of 1 to 10. Few patients (12.2%) contacted the GP with questions about their statin intake, in all cases regarding side effects of statin therapy. Half of the patients (48.6%) reached the LDL target value that the GP considered best for the patient. GPs’ most important reasons to deviate from the LDL target value recommended in the guideline were (in descending order of frequency) concerns about side effects due to a dose increase, patient’s non-adherence, and unhealthy lifestyle.

### Comparison between adherent and non-adherent patients

**Fig. 1:**
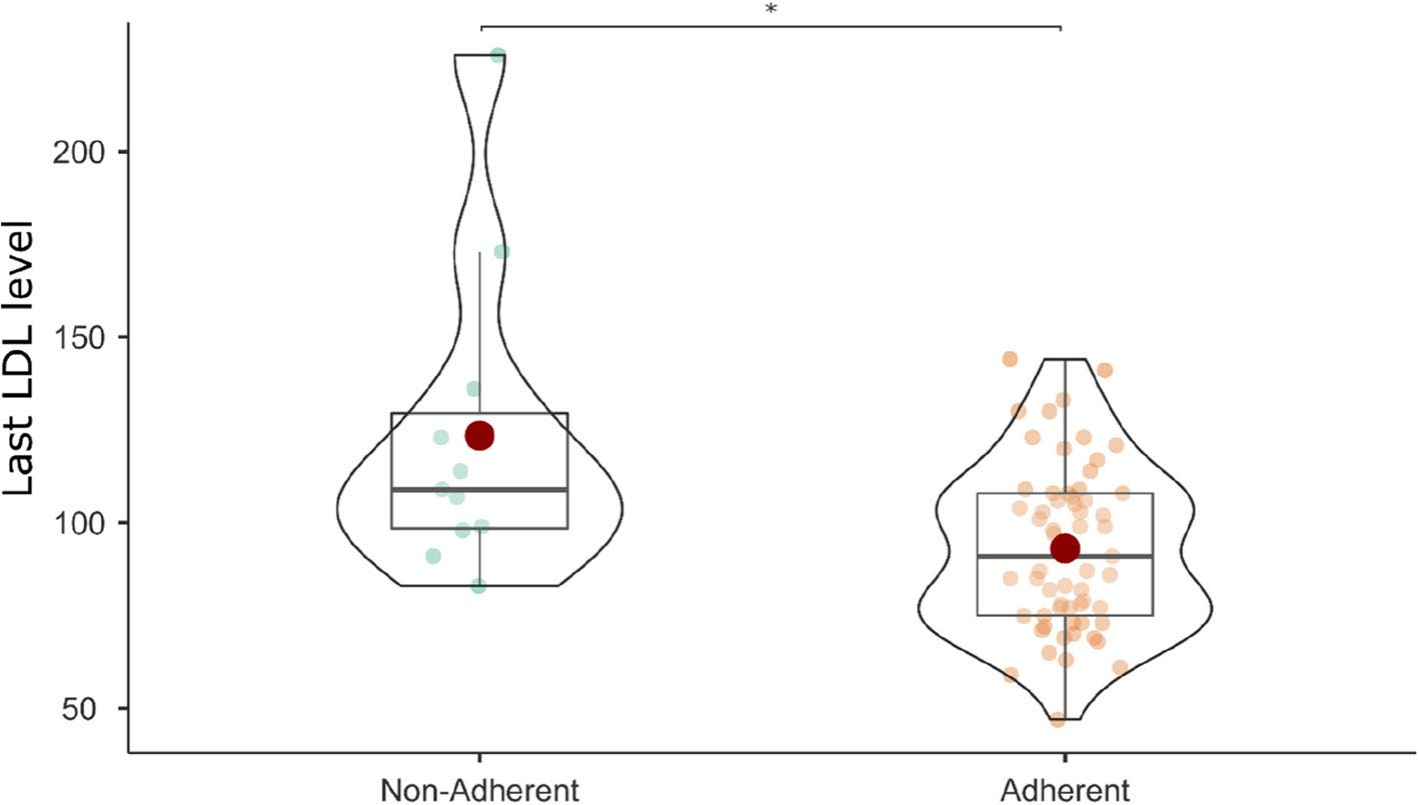
Differences in most recent LDL level (mg/dl) between adherent and non-adherent patients with CAD

The adherent patient group (*n* = 62) was further compared with the non-adherent patient group (*n* = 12). Non-adherent patients were significantly more likely to be employed (41.7%) than adherent patients (11.3%; χ2(1) = 6.828, *p* = 0.009, φ = -0.304). In addition, the most recent LDL level was lower in adherent patients (adherent = 93.08 mg/dl, non-adherent = 123.55 mg/dl; *F*(1, 70) = 12.959, *p* < 0.001, η_p_^2^ = 0.156; Fig. 1).

However, GPs’ assessment of patient's adherence did not differ between the groups classified as adherent and as non-adherent based on MPR. All results of the group comparison are presented in Table 1.

**Table 1:** Comparison of the adherent and non-adherent patient group

	**Adherent (*n* = 62)** **n (% as applicable)**	**Non-adherent (*n* = 12)** **n (% as applicable)**	***p***	**Effect size**
***Patient reports***				
Gender (Male/Female)	42/20 (67.7%/32.3%)	8/4 (66.7%/33.3%)	.593	-.008
Age	71.8 ± 10.2	67.8 ± 13.7	.251	.018
BMI	27.7 ± 4.2	28.9 ± 6	.414	.010
Partner (No/Yes)	20/42 (32.3%/67.7%)	6/6 (50%/50%)	.239	.137
Children (No/Yes)	9/53 (14.5%/85.5%)	1/11 (8.3%/91.7%)	.489	-.067
Education (Primary/ Secondary/Tertiary)	25/29/7 (41%/47.5%/11.5%)	7/5/0 (58.3%/41.7%)	.343	.171
Employed (No/Yes)	55/7 (88.7%/11.3%)	7/5 (58.3%/41.7%)	**.009**	-.304
Smoker (No/Yes)	47/15 (75.8%/24.2%)	9/3 (75%/25%)	.604	-.007
Alcohol consumption (No/Yes)	26/36 (41.9%/58.1%)	3/9 (25%/75%)	.221	-.128
Nursing degree (No/Yes)	56/6 (90.3%/9.7%)	11/1 (91.7%/8.3%)	1.000	.017
Knowledge about statin intake (No/Yes)	7/55 (11.3%/88.7%)	0/12(0/100%)	.221	-.142
Side effects (No/Yes)	50/10 (83.3%/16.7%)	10/2 (83.3/16.7%)	1.000	0
***Patient records***				
Type of CAD (Non-interventional/Interventional)	12/41 (22.6%/77.4%)	4/8 (33.3%/66.7%)	.470	.096
Most recent LDL value	93.1 ± 22	123.6 ± 42	** < .001**	.156
LDL target value in the last 12 months reached (No/Yes, at least once)	49/12 (80.3%/19.7%)	11/0 (100%/0%)	.192	.190
LDL target value ever reached (No/Yes, at least once)	43/17 (71.7%/28.3%)	11/0 (100%/0%)	.055	.240
No. of interventions relevant to the coronary artery in the last 12 months				
0	27 (43.5%)	10 (83.3%)	.076	.339
1	10 (16.1%)	0		
2	8 (12.9%)	2 (16.7%)		
3	4 (6.5%)	0		
> 3	13 (21%)	0		
Hospital admission in the last 12 months (No/Yes)	53/9 (85.5%/14.5%)	12/0 (100%/0%)	.339	.164
***GPs’ assessments***				
GPs’ assessment of patient adherence	8.3 ± 1.7	8.6 ± 1.3	.533	.005
Patient asked questions about statin intake (No/Yes)	55/7 (88.7%/11.3%)	10/2 (83.3%/16.7%)	.633	-.061
Patient is reaching target LDL (No/Yes)	29/32 (47.5%/52.5%)	8/4 (66.7%/33.3%)	.345	.142

Categorial variables were analyzed by using chi-square or Fisher's exact tests and estimated effect sizes were reported using Phi (φ) or Cramer's V. Continuous variables were analyzed by using ANOVAs and estimated effect sizes were reported using partial eta squared (η_p_^2^). Education was assessed by using the CASMIN educational classification

## Discussion

In this study, the statin adherence of patients in the DMP-CAD was assessed using patient questionnaires, GPs’ assessments, and prescription data. Overall, most patients exhibited a high level of statin adherence. However, GPs’ ratings of assumed adherence did not differ between the adherent and the non-adherent group, indicating potential difficulties. In addition, the target LDL-C value was rarely reached despite high adherence. Although the physicians were aware of the deviation from the guideline, they often did not adjust the statin therapy, mostly to avoid side effects.

A meta-analysis reported 54% statin adherence in patients with cardiovascular diseases [6], far below the 83% adherence value measured in the present study, indicating an increased patient adherence in structured programs [18]. A positive effect of the DMP on medication and especially statin adherence has also been shown in other studies comparing patients enrolled and not enrolled in the DMP [10, 11].

The study population had similar sociodemographic characteristics as the DMP CHD cohort North Rhine during this period. There, the age was 72.8 ± 11.2 years (study cohort: 71.1 ± 10.8) and the proportion of male patients was 64.9% (study cohort: 67.6%) [19]. The age of the study participants was therefore only slightly lower and the proportion of men only slightly higher than in the DMP CAD North Rhine. Also, in the DMP CAD North Rhine, a similarly high guideline-compliant statin prescription rate is observed as in the study: in the study, the rate is 87%, in the DMP 84.5% [19]. The statin prescription rate in DMP CAD North Rhine has increased significantly in recent years. In the DMP CAD North Rhine, a total of 61.6% of all patients achieve an LDL-C < 100 mg/dl (female ≥ 76 years: 50.6%, male ≥ 76 years: 67.5%). An LDL-C < 70 mg/dl is achieved by 22.4% of all patients [19]. In the DMP CAD North Rhine, statin prescription correlates with the male gender of the patients, specialist care and high continuity of participation in the DMP CAD [20].

Like other studies, our cohort showed that a good adherence is not necessarily associated with optimal LDL levels in patients. In a study investigating the effects of regular statin use on LDL levels, it was found that many patients with CAD do not reach the target LDL level despite regular statin use [5]. Consequently, LDL values should not be used as the only indicator to determine patient adherence.

Not reaching the target LDL value can also be caused by an insufficient efficacy or dosage of the medication. Moreover, statin adherence also depends on the statin dosage, which has been shown by a comparison of a low-dose with a high-dose group; the side effect rate was higher in the high-dose group (8.1% vs. 5.8%) and resulted more often in therapy discontinuation (7.2% vs. 5.3%) [5]. In line, GPs in our study may want to avoid higher doses of statins to avoid reducing their patients' adherence due to side effects. A feasible option would be to switch the statin to a more effective preparation where a lower dose would be sufficient to achieve the therapeutic goal, or to combine it with a cholesterol uptake inhibitor such as ezetimibe.

Further, we found that GPs did not reliably identify non-adherence in their patients. Similarly, other studies showed that GPs often estimate the prevalence of non-adherence incorrectly and define non-adherence differently [21]. For a better assessment of adherence, GPs could more frequently ask their patients about medication intake and possible side effects. In addition, the current prescription programs provide indications whether patients regularly collect their prescriptions in case of continuous intake. To increase time efficiency of adherence checks, our data could potentially provide information on which patients are most likely to be non-adherent. For instance, in our study, non-adherent patients were more likely to be employed. Possible explanations are provided by studies showing that occupational stress was associated with lower statin adherence [22]. Due to the small number of participants in our study, these are only preliminary indications of a potential patient pattern in which the GP should monitor adherence.

### Strengths and limitations

Our study is the first considering information provided by the patient, the GP, as well as data from the patient records within the DMP-CAD. The participation rate was 61.2%, indicating a potential bias towards participation of patients with better adherence.

Another limitation is the small sample size and the selection of three rather rural and spatially close GP practices. Due to the pandemic, the DMP-CAD was temporarily suspended, challenging the recruitment of patients and practices during the study period. Conclusions can only be drawn to a limited extent about patients in the DMP-CAD in Germany and should be replicated in future studies.

## Conclusion

In this study, LDL targets were often not reached despite good statin adherence. GPs should take advantage of the high adherence of their patients to achieve the guideline recommendations for LDL management more frequently by adjusting the agents and/or dose. Larger studies could provide guidance about which patient groups should be particularly monitored for statin adherence.

### Supplementary Information

**Additional file 1: Supplementary material S1.** Questionnaire for the collection of patient data and statin adherence a) in translated version and b) in the original. The questionnaire is divided into a patient questionnaire, a survey of data from the patient file, and a physician questionnaire.

## Data Availability

Data generated or analyzed during the study are available from the corresponding author by reasonable request.
