# Supplementary material for: Statin adherence in patients enrolled in the disease management program for coronary artery disease – comparison between patients’ and general practitioners’ self-reports and patient records
Source: Res Health Serv Reg. 2023 Sep 6;2:13. doi: 10.1007/s43999-023-00029-3 (PMC11281732; doi:10.1007/s43999-023-00029-3)
Supplement: Supplementary file 1 — Additional file 1: Supplementary material S1. Questionnaire for the collection of patient data and statin adherence a) in translated version and b) in the original. The questionnaire is divided into a patient questionnaire, a survey of data from the patient file, and a physician questionnaire. [file 43999_2023_29_MOESM1_ESM.docx]

**Supplementary material S1.** Questionnaire for the collection of patient data and statin adherence a) in translated version and b) in the original. The questionnaire is divided into a patient questionnaire, a survey of data from the patient file, and a physician questionnaire.

a) English version

**Patient Questionnaire:**

Date: _ _. _ _ ._ _ _ _ Practice no. _ _

Patient number: _ _ _

When answering the questions, please try to use the full breadth of answer options and do not omit any question if possible.

1. Gender

- Male
- female

1. Your age : _____ years
2. Your size : _____ cm
3. Your current weight: _____ kg
4. Marital status
   - Single
   - Married
   - Permanent relationship
   - Divorced
   - Widowed
5. Do you have children?
   - No

Yes

If yes, how many? : _____

1. Your highest level of education
   - No school diploma
   - Elementary school
   - Secondary school leaving certificate
   - University of Applied Sciences certificate
   - General qualification for university entrance
   - Academic degree
   - Vocational school
   - Other : _______________________________________
2. Are you currently employed?
   - Yes, full day
   - Yes, (about) half-day
   - Pensioner
   - No, in education
   - No, Housewife/Househusband
   - No, unemployed
   - No, other _______________
3. Are you a smoker?
   - No
   - Yes
4. If yes, how many cigarettes do you smoke per day?
   - 1-5
   - 5-15
   - 15-25
   - >25
5. How often do you drink alcohol?
   - Never
   - Once a month
   - 2-4 times per month
   - 2-4 times per week
   - More than 4 times a week
6. If you drink alcohol in a day, how many alcoholic beverages do you typically drink? (One glass of alcohol is equivalent to 0.33l beer, 0.15l wine/sparkling wine or 0.04l spirits)

- 1-2
- 3-4
- 5-6
- 7-8
- 9 or more

1. Do you have a care degree?
   - No
   - Yes

If yes, which one: _____ (1-5)

1. Are you taking any additional medications/ herbal substances/ dietary supplements **not prescribed by your doctor**?
   - No
   - Yes

If yes, which?

____________________________________________________________________________________________________________________________________________________________________________________________________________

1. Are you taking a cholesterol-lowering drug/ lipid-lowering drug (statin)?
   - No
   - Don't know
   - Yes
2. Have cholesterol-lowering drugs/ lipid-lowering drugs (statin) ever caused you side effects?
   - No
   - Yes

If so, in what way?

- - Headache
  - Digestive problems
  - Muscle pain without muscle weakness (myalgia)
  - Muscle pain associated **with** muscle weakness (myopathy)
  - Other side effects: __________________________________

1. **Only if side effects have already occurred:**

If side effects occurred due to lipid-lowering/ statin use, how often and how severe were they?

- - Every day
  - Several times a week
  - Once a week
  - Several times a month
  - Rarer

1. How severe are/were your side effects from cholesterol-lowering drugs/ lipid-lowering drugs (statin) on a scale of 0-10

(From 0 no side effects at all to 10 most severe side effects): _____

**Data from patient record:**

**As of this parameter, we consider the last 12 months as the defined study period**

1. Type of CHD (which one?)

- 1-vessel CHD
- 2-vessel CHD
- 3-vessel CHD
- PCI
- PCI + Stent
- CABG

1. If LDL serum history available
   - What was the last measured LDL level [mg/dl]: _____
   - Number of measurements with LDL <70 mg/dl in the last 12 months: ____
   - Was an LDL <70 mg/dl achieved once? (Yes/No): _____
2. How many drug prescriptions for statins were there during the relevant study period? (Consideration of mode of intake and dosage)
   - ___ x N1 (20 Tablets)
   - ___ x N2 (50 Tablets)
   - ___ x N3 (100 Tablets)

What drug dose and mode / were half tablets taken?

___________________________________________________________________

Adherence calculation result/ Medication Posession Ratio: _________

1. How many relevant events (diagnostic + coronary-related interventions) occurred during the defined study period?
   - 0
   - 1
   - 2
   - 3
   - > 3
2. Was there any emergency inpatient treatment for CHD during the defined study period?
   - No
   - Yes

**Physician questionnaire:**

**Please only have the doctor fill out**

**As of this parameter, we consider the last 12 months as the defined study period**

1. How do you perceive the patient's adherence? (0: very weak; 10 very strong)

0 10

1. Has the patient approached you with questions about their own statin intake?
   - No
   - Yes
2. If yes, why?

______________________________________________________________

______________________________________________________________

______________________________________________________________

1. In your opinion, does the patient reach the target LDL value?
   - No
   - Yes
2. If not, why not? ________________________________________________________________________________________________________________________________________________________________________________________________________________________________________________________________________________
3. Please enclose the patient's medication schedule

b) original version

**PatientInnenfragebogen:**

Datum: _ _. _ _ ._ _ _ _ Praxis Nr. _ _

PatientInnennummer: _ _ _

Versuchen Sie bitte, bei der Beantwortung der Fragen die gesamte Breite der Antwortoptionen zu nutzen, und lassen Sie nach Möglichkeit keine Frage aus.

1. Geschlecht

- Männlich
- Weiblich

1. Ihr Alter : _____ Jahre
2. Ihre Größe : _____ cm
3. Ihr aktuelles Gewicht : _____ kg
4. Familienstand
   - Ledig
   - Verheiratet
   - Feste Partnerschaft
   - Geschieden
   - Verwitwet
5. Haben Sie Kinder?
   - Nein
   - Ja

Wenn ja, wie viele? : _____

1. Ihr höchster Bildungsabschluss
   - Kein Schulabschluss
   - Hauptschule/Volksschule
   - Mittlere Reife/Realschule
   - Fachhochschule
   - Abitur/Allg. Hochschulreife
   - Akademischer Abschluss
   - Berufsschule/Berufsfachschule
   - Sonstiges : _______________________________________
2. Sind Sie zurzeit erwerbstätig?
   - Ja, ganztags
   - Ja, (ungefähr) halbtags
   - Rentner
   - Nein, in Ausbildung
   - Nein, Hausfrau/Hausmann
   - Nein, arbeitslos/erwerbslos
   - Nein, anderes _______________
3. Sind Sie Raucher?
   - Nein
   - Ja
4. Wenn ja, wie viele Zigaretten rauchen Sie pro Tag?
   - 1-5
   - 5-15
   - 15-25
   - >25
5. Wie oft trinken Sie Alkohol?
   - Nie
   - Einmal pro Monat
   - 2-4 mal pro Monat
   - 2-4 mal pro Woche
   - Öfter als 4 mal Woche
6. Wenn Sie an einem Tag Alkohol trinken, wie viele alkoholhaltige Getränke trinken Sie dann typischerweise? Ein Glas Alkohol entspricht 0,33l Bier, 0,15l Wein/Sekt oder 0,04l Spirituosen)

- 1-2
- 3-4
- 5-6
- 7-8
- 9 oder mehr

1. Haben Sie einen Pflegegrad?
   - Nein
   - Ja

Wenn ja, welchen: _____ (1-5)

1. Nehmen Sie zusätzliche, **nicht vom Arzt verordnete,** Medikamente/ pflanzliche Stoffe/ Nahrungsergänzungsmittel ein?
   - Nein
   - Ja

Wenn Ja, welche ?

____________________________________________________________________________________________________________________________________________________________________________________________________________

1. Nehmen Sie einen Cholesterinsenker/ Fettsenker (**Statin**) ein?
   - Nein
   - Weiß ich nicht
   - Ja
2. Haben Ihnen die Cholesterinsenker/ Fettsenker (**Statin**) schon einmal Nebenwirkungen bereitet?
   - Nein
   - Ja

Wenn ja, in welcher Form?

- - Kopfschmerzen
  - Verdauungsbeschwerden
  - Muskelschmerzen **ohne** Muskelschwäche (Myalgie)
  - Muskelschmerzen in Verbindung **mit** Muskelschwäche (Myopathie)
  - Andere Nebenwirkungen: __________________________________

1. **Nur falls Nebenwirkungen schon mal aufgetreten sind:**

Falls Nebenwirkungen durch Fettsenkereinnahme/ Statineinnahme aufgetreten sind, wie häufig und wie stark waren diese?

- - Jeden Tag
  - Mehrmals die Woche
  - Einmal die Woche
  - Mehrmals im Monat
  - Seltener

1. Wie groß sind/waren Ihre Nebenwirkungen durch Cholesterinsenker/ Fettsenker (**Statin**) auf einer Skala von 0-10

(Von 0 gar keine Nebenwirkungen bis 10 stärkste Nebenwirkungen): _____

**Daten aus PatientInnenakte:**

**Ab diesem Parameter betrachten wir die letzten 12 Monate als definierten Untersuchungszeitraum**

1. Art der KHK (Welche?)

- 1-Gefäß-KHK
- 2-Gefäß-KHK
- 3-Gefäß-KHK
- PTCA
- PTCA+ Stent
- ACVB
- ACB

1. Falls LDL-Serumverlauf vorhanden
   - Wie hoch war der letzte gemessene LDL-Wert [mg/dl]: _____
   - Anzahl der Messungen mit LDL <70 mg/dl in den letzten 12 Monaten: ____
   - Wurde ein LDL <70 mg/dl einmalig erreicht? (Ja/Nein): _____
2. Wie viele Medikamentenverordnungen von Statinen gab es im relevanten Untersuchungszeitraum? (Berücksichtigung von Einnahmemodus und Dosierung)
   - ___ x N1 (20 Tabletten)
   - ___ x N2 (50 Tabletten)
   - ___ x N3 (100 Tabletten)

Welche Medikamentendosis und -modus / wurden halbe Tabletten eingenommen? ____________________________________________________________________

Ergebnis der Adhärenzberechnung/ Medication Posession Ratio: _______________

1. Wie viele relevante Ereignisse (diagnostische + koronarrelevante Interventionen) gab es in dem definierten Untersuchungszeitraum?
   - 0
   - 1
   - 2
   - 3
   - > 3
2. Gab es im definierten Untersuchungszeitraum eine stationäre notfallmäßige Behandlung aufgrund der KHK?
   - Nein
   - Ja

**Arztfragebogen:**

**Bitte nur vom Arzt ausfüllen lassen**

**Ab diesem Parameter betrachten wir die letzten 12 Monate als definierten Untersuchungszeitraum**

1. Wie nehmen Sie die Adhärenz des Patienten/ der Patientin wahr? (0: sehr schwach; 10 sehr stark)

0 10

1. Ist der Patient/ die Patientin mit Fragen über die eigene Statinaufnahme auf Sie zugekommen?
   - Nein
   - Ja
2. Wenn Ja warum?

______________________________________________________________

______________________________________________________________

______________________________________________________________

1. Erreicht der Patient/ die Patientin aus Ihrer Sicht den Ziel-LDL-Wert?
   - Nein
   - Ja
2. Wenn Nein, warum nicht? ________________________________________________________________________________________________________________________________________________________________________________________________________________________________________________________________________________
3. Bitte legen Sie den Medikationsplan des Patienten/ der Patientin bei
